# Strategies to Mitigate Cisplatin-Induced Ototoxicity: A Literature Review of Protective Agents, Mechanisms, and Clinical Gaps

**DOI:** 10.3390/audiolres15020022

**Published:** 2025-02-27

**Authors:** Alexandru Orasan, Mihaela-Cristina Negru, Anda Ioana Morgovan, Razvan Claudiu Fleser, Daniela Sandu, Adrian Mihail Sitaru, Alexandru-Catalin Motofelea, Nicolae Constantin Balica

**Affiliations:** 1 ENT Department, “Victor Babes” University of Medicine and Pharmacy, Eftimie Murgu Square No. 2, 300041 Timisoara, Romania; alexandru.orasan@umft.ro (A.O.); anda.morgovan@umft.ro (A.I.M.); balica@umft.ro (N.C.B.); 2 Otorhinolaryngology Department, “Iuliu Hatieganu” University of Medicine and Pharmacy, 400347 Cluj-Napoca, Romania; fleser_razvan_claudiu@elearn.umfcluj.ro; 3 OncoHelp Cancer Centre, Radiation Oncology Department, “Victor Babes” University of Medicine and Pharmacy, Str. Rusu Sireanu nr. 34 Timisoara, 300041 Timisoara, Romania; daniela.sandu@umft.ro; 4 Department of Pediatric Surgery, “Louis Turcanu” Emergency Clinical Hospital for Children, Iosif Nemoianu Street 2, 300011 Timisoara, Romania; adrian.sitaru@umft.ro; 5 Center for Molecular Research in Nephrology and Vascular Disease, Faculty of Medicine, “Victor Babeș” University of Medicine and Pharmacy, 300041 Timișoara, Romania; alexandru.motofelea@umft.ro

**Keywords:** cisplatin, ototoxicity, chemotherapy, antioxidants, nanoparticles, apoptosis

## Abstract

Background: Cisplatin, a widely used chemotherapeutic agent, is associated with significant ototoxicity, leading to progressive and irreversible sensorineural hearing loss in up to 93% of patients. Cisplatin generates reactive oxygen species (ROS) in the cochlea, activating apoptotic and necroptotic pathways that result in hair cell death. Inflammatory processes and nitrative stress also contribute to cochlear damage. Methods: This literature review was conducted to explore the mechanisms underlying cisplatin-induced ototoxicity and evaluate protective strategies, including both current and emerging approaches. A structured search was performed in multiple scientific databases, including PubMed and ScienceDirect, for articles published up to November 2024. Results: Current otoprotective strategies include systemic interventions such as antioxidants, anti-inflammatory agents, and apoptosis inhibitors, as well as localized delivery methods like intratympanic injection and nanoparticle-based systems. However, these approaches have limitations, including potential interference with cisplatin’s antitumor efficacy and systemic side effects. Emerging strategies focus on genetic and biomarker-based risk stratification, novel otoprotective agents targeting alternative pathways, and combination therapies. Repurposed drugs like pravastatin also show promise in reducing cisplatin-induced ototoxicity. Conclusions: Despite these advancements, significant research gaps remain in translating preclinical findings to clinical applications and developing selective otoprotective agents that do not compromise cisplatin’s efficacy. This review examines the mechanisms of cisplatin-induced ototoxicity, current otoprotective strategies, and emerging approaches to mitigate this adverse effect.

## 1. Introduction

Cisplatin, a platinum-based chemotherapeutic agent, is widely used in treating various solid tumors due to its potent anticancer activity. It is particularly effective against testicular, ovarian, head and neck, bladder, lung, and cervical cancers, as well as melanoma and lymphomas [1,2]. The rate of head and neck cancer (HNC) is expected to increase by 30% by 2030, highlighting the growing need for effective treatments and comprehensive diagnostic approaches. However, there are many similarities between the symptomatology of benign and malignant diagnoses, underscoring the importance of a protocolized examination since the absence of adenopathy does not exclude a malignant diagnosis, which can affect prognosis [3]. Cisplatin’s remarkable contribution to cancer treatment is most evident in testicular cancer, where it has significantly improved response and survival rates. Prior to cisplatin’s introduction, testicular tumors had response rates of approximately 50%, but cisplatin-based therapy now cures the majority of patients with advanced stage disease [4]. Interestingly, while cisplatin has clearly improved response rates for testicular, ovarian, and small cell lung tumors, its impact on overall survival has been marginal in most cases [5]. This limitation is often attributed to the development of drug resistance in tumor cells. Its use is associated with significant side effects, particularly ototoxicity—progressive and irreversible sensorineural hearing loss [6,7]. Up to 93% of patients receiving cisplatin chemotherapy may develop hearing impairment, which can severely impact quality of life in cancer survivors [6,8]. Interestingly, the severity of cisplatin-induced ototoxicity is related to factors like age and cumulative dose, with younger patients and higher total doses leading to more severe hearing loss [9]. While cisplatin affects hearing, Mosalaei et al. found no statistically significant effect on the sense of smell at doses causing hearing impairment, highlighting the specificity of its ototoxic effects [10]. Despite its ototoxic potential, cisplatin remains a crucial component of cancer treatment due to its high efficacy. Currently, there is no approved treatment or prevention for cisplatin-induced ototoxicity in adult patients [6,11]. In the pediatric population, sodium thiosulfate (Pedmark) received approval in September 2022 in the USA for reducing the risk of ototoxicity associated with cisplatin in patients aged 1 month and older with localized, non-metastatic solid tumors [12].

A systematic review has identified sodium thiosulfate as the most promising intervention for preventing cisplatin-induced ototoxicity in adult cancer patients. However, current interventions have shown only mild benefits [13].

This presents a significant challenge in balancing the benefits of cisplatin therapy against its potential long-term effects on hearing, especially in pediatric patients where the impact on quality of life can be particularly profound [9,11]. Cisplatin remains a cornerstone in cancer chemotherapy, particularly for solid tumors. Its widespread use is due to its proven efficacy in improving response rates and, in some cases, survival rates. However, the challenges of drug resistance and side effects have led to ongoing research into combination therapies, novel drug delivery systems, and the development of other platinum-based and non-platinum metal complexes to enhance its therapeutic potential and mitigate its limitations [14,15]. The aim of this review is to provide a comprehensive evaluation of cisplatin-induced ototoxicity, focusing on its molecular mechanisms, existing protective strategies, and emerging therapeutic approaches. A key research gap lies in the translation of promising preclinical interventions into clinically viable therapies that selectively prevent ototoxicity without compromising cisplatin’s antitumor efficacy. This gap is further compounded by the limited understanding of inter-individual variability in ototoxicity risk, driven by genetic, demographic, and treatment-related factors. By addressing these challenges, this review seeks to propose targeted, evidence-based strategies to mitigate cisplatin-induced ototoxicity while preserving its chemotherapeutic effectiveness.

## 2. Material and Methods

This literature review was conducted to explore the mechanisms underlying cisplatin-induced ototoxicity and evaluate protective strategies, including current and emerging approaches. A structured search was performed in multiple scientific databases, including PubMed and ScienceDirect, for articles published up to November 2024. The search utilized terms such as “cisplatin”, “ototoxicity”, “hearing loss”, “otoprotective agents”, and “oxidative stress”. Boolean operators and Medical Subject Heading (MeSH) terms were employed to refine the results and ensure relevance. In addition to database searches, reference lists of key articles were manually reviewed to identify additional relevant studies.

The selection of articles followed a structured inclusion and exclusion process. Studies were included if they addressed the mechanisms of cisplatin-induced ototoxicity or evaluated otoprotective interventions. Eligible articles encompassed reviews, clinical studies, and preclinical research that provided mechanistic insights or discussed interventions aimed at mitigating cisplatin-induced hearing loss. Articles not focused on cisplatin-related ototoxicity or lacking substantial data on mechanisms or protective strategies were excluded. Only articles published in English or with accessible English translations were considered for this review.

The study selection process involved an initial screening of titles and abstracts to assess their relevance. Full-text reviews were then conducted to confirm the eligibility of the selected articles. Two independent reviewers performed the screening and selection, resolving discrepancies through consensus or, if necessary, by involving a third reviewer.

Key information included study design, mechanisms of cisplatin-induced ototoxicity, protective strategies, and their outcomes. Mechanistic data were categorized into oxidative stress, apoptosis, and emerging pathways, while otoprotective interventions were grouped into systemic and localized approaches. Outcomes were analyzed with a focus on their effectiveness in preventing ototoxicity and their potential impact on cisplatin’s chemotherapeutic efficacy.

The synthesis of findings was conducted narratively. Mechanisms of cisplatin-induced ototoxicity were outlined, highlighting oxidative stress and other cellular pathways. Protective strategies were evaluated, emphasizing their strengths, limitations, and clinical applicability. Emerging therapeutic approaches were also discussed, with attention to the translation of preclinical findings to clinical settings. Throughout the review, research gaps and areas requiring further investigation were identified, providing a foundation for future studies.

## 3. Results

### Mechanisms of Cisplatin-Induced Ototoxicity

Reactive oxygen species (ROS) play a significant role in cochlear cell damage and apoptosis, contributing to various forms of hearing loss. ROS are highly reactive molecules that can oxidize proteins, lipids, and DNA, potentially leading to cell death through apoptosis or necrosis [16]. In the cochlea, which is particularly vulnerable to oxidative stress, ROS generation is common in many conditions. This can result in cell death, making it a critical area of study for hearing loss prevention and treatment [17]. The cochlea is particularly vulnerable to oxidative stress, which can be induced by various factors such as noise exposure, ototoxic drugs, and aging. These stressors can trigger the production of ROS, leading to cochlear cell damage and ultimately hearing loss [18]. Interestingly, while ROS are generally considered harmful, they also serve as essential intracellular second messengers for certain cytokines and growth factors, highlighting their complex role in cellular processes [16]. To combat the detrimental effects of ROS, the cochlea employs various protective mechanisms. One such mechanism involves corticotropin-releasing factor (CRF) signaling, which has been shown to protect against aminoglycoside-induced ROS production and activation of cell death pathways [17]. Additionally, antioxidant systems play a crucial role in neutralizing ROS, although their effectiveness may be limited during periods of high ROS production [19]. Understanding these protective mechanisms and the intricate balance between ROS production and elimination is essential for developing strategies to prevent and treat hearing loss caused by oxidative stress in the cochlea.

Cisplatin exposure activates both apoptotic and necroptotic pathways in the cochlea, leading to hair-cell death and hearing loss. Cisplatin generates reactive oxygen species (ROS) in the inner ear, which can trigger cell death pathways [20]. These ROS activate the c-Jun N-terminal kinase (JNK) and p38 mitogen-activated protein kinase (MAPK) pathways, ultimately inducing hair cell apoptosis [20]. Interestingly, while ex vivo studies suggest that only apoptosis contributes to cisplatin ototoxicity, in vivo research indicates that both apoptosis and necroptosis are involved in cisplatin-induced cochlear damage [21]. Necroptosis, a programmed form of necrosis, involves receptor-interacting protein kinase (RIPK) 1 and RIPK3 [21]. This finding contradicts the traditional view that necrosis in cisplatin ototoxicity is solely a passive process. Recent studies have also identified additional cell death pathways activated by cisplatin in the cochlea, including ferroptosis [22]. The involvement of multiple cell death pathways highlights the complexity of cisplatin ototoxicity and suggests that targeting a single pathway may not be sufficient for complete otoprotection. Understanding these diverse mechanisms provides new avenues for developing therapeutic strategies to prevent or mitigate cisplatin-induced hearing loss, such as the use of antioxidants, caspase inhibitors, and necroptosis inhibitors [21,22].

Recent research has highlighted the involvement of emerging pathways in cisplatin-induced ototoxicity, including autophagy and nitrative stress. Autophagy, traditionally considered a survival mechanism, has been shown to play a dual role in cisplatin ototoxicity. Early upregulation of autophagy via the class III PI3K pathway exerts cytoprotective effects, while later increases in autophagy through mTOR pathway suppression can induce cell death [23]. This suggests that modulating the autophagic pathway could be a potential strategy against cisplatin-induced hearing loss. Nitrative stress, particularly induced by S-nitrosylation of cochlear proteins, has been implicated in cisplatin ototoxicity. Studies have shown that cisplatin treatment leads to increased S-nitrosylation of at least three cochlear proteins in the organ of Corti, stria vascularis, and spiral ganglions, which are known targets of cisplatin toxicity.

Although direct evidence identifying specific S-nitrosylated proteins remains limited, available proteomic and immunohistochemical data suggest that nitrative modifications, including S-nitrosylation, contribute to cochlear dysfunction. For example, LIM Domain Only 4 (LMO4) is notably downregulated in response to cisplatin treatment, with its decreased expression correlating with increased ototoxicity severity [24]. Additionally, the localization of nitrotyrosine-bearing proteins within outer hair cells indicates that nitrative stress—potentially encompassing S-nitrosylation—compromises protein function in the cochlea [25]. Furthermore, the observed downregulation of Ras-related proteins, such as Rab-2A and Rab-6A, in cisplatin-treated cochlear tissues supports the hypothesis that nitrative modifications disrupt intracellular signaling pathways critical for hair cell survival [26]. Collectively, these findings underscore the likely role of S-nitrosylation as one component of the nitrative stress response that exacerbates oxidative damage and promotes apoptotic cascades in cisplatin-induced ototoxicity. Further targeted research is required to directly identify and characterize S-nitrosylated cochlear proteins, which could ultimately lead to novel therapeutic strategies aimed at mitigating cisplatin-induced hearing loss [27].

Importantly, the inhibition of peroxynitrite formation by Trolox attenuated both S-nitrosylation and hearing loss, suggesting a crucial role for this modification in mediating cisplatin’s ototoxic effects [28]. Parthanatos, a PARP-1-dependent form of programmed cell death, has been linked to oxidative stress-induced cell death in various tissues [29,30]. While not directly studied in the context of cisplatin ototoxicity, the involvement of oxidative stress and PARP-1 activation in cisplatin-induced hearing loss suggests that parthanatos may play a role in this process. Further research is needed to elucidate the specific contribution of parthanatos to cisplatin ototoxicity and its potential as a therapeutic target. These emerging pathways provide new insights into the complex mechanisms underlying cisplatin-induced hearing loss. Understanding the interplay between autophagy, nitrative stress, and potentially parthanatos could lead to the development of novel protective strategies against cisplatin ototoxicity, ultimately improving the quality of life for cancer patients undergoing chemotherapy. Table 1 highlights the main mechanisms through which cisplatin induces ototoxicity.

## 4. Current Otoprotective Strategies

### 4.1. Systemic Interventions

Antioxidants have shown promise in mitigating cisplatin-induced nephrotoxicity, which affects approximately one-third of patients receiving this treatment [31]. Oxidative stress is a significant mechanism in cisplatin nephrotoxicity, stimulating apoptosis, inflammation, mitochondrial damage, and endoplasmic reticulum stress. The administration of antioxidants could be a suitable approach for preventing these adverse effects [31]. Studies have demonstrated that radical scavengers like α-tocopherol and N-N’-diphenyl-p-phenylenediamine can decrease blood urea nitrogen induced by cisplatin, suggesting that the toxic effects of cisplatin may be related to free radical-induced damage [32]. Additionally, trans-3,3′,5,5′-tetrahydroxy-4′-methoxystilbene and resveratrol have shown significant inhibition of protein carbonylation, reduction of thiol group oxidation, and decreased lipid peroxidation in blood platelets treated with platinum compounds [33]. However, it is important to note that while antioxidants may help reduce cisplatin-induced toxicity, there are concerns about potential interference with cisplatin’s antitumor effect. The anticancer properties of platinum compounds are primarily attributed to their ability to form covalent adducts on DNA [34,35]. Antioxidants could potentially interfere with this mechanism, potentially reducing the drug’s efficacy. Therefore, further research is needed to determine the optimal balance between reducing toxicity and maintaining antitumor activity when combining antioxidants with cisplatin treatment. In conclusion, while antioxidants show promise in mitigating cisplatin-induced toxicity, careful consideration must be given to their potential impact on the drug’s antitumor efficacy.

Apoptosis inhibitors have shown promising results in reducing cochlear cell death and protecting against hearing loss in various studies. Tabuchi et al. showed that X-linked inhibitor of apoptosis protein (XIAP) decrease caspase-3 activation and hair cell loss during gentamicin ototoxicity, acting as part of a protective response [36]. XIAP inhibitors increased gentamicin-induced hair cell loss, indicating XIAP’s protective role. Antioxidants like L-N-Acetylcysteine (L-NAC) have demonstrated effectiveness in reducing radiation-induced apoptosis in cochlear cells by decreasing reactive oxygen species (ROS) generation [37]. L-NAC increased cell viability and diminished apoptosis after irradiation. Apaf-1 inhibitors have shown potential in preventing cell and tissue damage in animal models of apoptotic damage by decreasing cytochrome c release and apoptosome-mediated activation of procaspase-9 [38]. This suggests Apaf-1 inhibition may have therapeutic potential for apoptosis-related diseases. Oral administration of antioxidant vitamins A, C, E, and magnesium (ACEMg) improved auditory function and increased survival of sensory outer hair cells after noise-induced hearing loss in rats [39]. ACEMg modulated the expression of antioxidant enzymes, diminished pro-apoptotic proteins, and increased anti-apoptotic Bcl-2 levels. In summary, various apoptosis inhibitors, including XIAP, antioxidants, and Apaf-1 inhibitors, have demonstrated efficacy in reducing cochlear cell death through different mechanisms. These findings highlight the potential of targeting apoptotic pathways for hearing protection and treatment of hearing loss.

Studies have demonstrated that inhibition of NF-κB activation or suppression of inflammatory mediators can attenuate hearing loss, highlighting the significance of inflammatory pathways in cisplatin-induced ototoxicity [40]. The NF-κB signaling cascade plays a central role in inflammation by regulating the release of pro-inflammatory mediators, including inducible nitric oxide synthase (iNOS) and tumor necrosis factor-alpha (TNF-α), which both contribute to cochlear damage and hearing loss [40]. Various compounds have shown potential in mitigating this inflammatory response by inhibiting NF-κB activation and reducing the production of pro-inflammatory cytokines. Notably, rhein, alantolactone, celastrol, and parthenolide have been identified as effective inhibitors of NF-κB signaling, thereby exerting otoprotective effects [41,42,43,44].

While targeting NF-κB activation appears to be a promising strategy, its role in immune homeostasis must be carefully considered. Some studies suggest that NF-κB plays a protective role in certain tissues by maintaining immune equilibrium and preventing excessive inflammatory damage [45]. This dual function highlights the complexity of inflammatory pathways and underscores the need for targeted approaches in treating cisplatin-induced hearing loss.

Cisplatin-induced activation of NF-κB and the subsequent release of inflammatory mediators such as TNF-α, interleukin-6 (IL-6), and cyclooxygenase-2 (COX-2) contribute to both cochlear cell death and nephrotoxicity [46]. Hyperin, a flavonoid compound, has been shown to reduce cisplatin-induced acute kidney injury by inhibiting NF-κB activation and suppressing inflammatory cytokines such as TNF-α, IL-1β, and IL-6 (Chao et al., 2016). Similarly, polysulfide and hydrogen sulfide donors have demonstrated protective effects against cisplatin nephrotoxicity by inhibiting NF-κB phosphorylation and preventing the degradation of IκBα, ultimately reducing the expression of pro-inflammatory factors [47]. These findings reinforce the notion that targeting NF-κB could be a viable therapeutic strategy to mitigate cisplatin-induced toxicity.

Interestingly, the relationship between NF-κB activation and cell death appears to be context-dependent. In hepatic cells, for instance, pretreatment with TNF-α or IL-1β has been found to activate NF-κB and prevent apoptosis under specific conditions [48]. This paradox suggests that while NF-κB inhibition may be beneficial in attenuating inflammatory damage in the cochlea, its broader physiological functions should be carefully evaluated.

Overall, the suppression of NF-κB activation and inflammatory mediators presents a compelling strategy for mitigating cisplatin-induced ototoxicity. Several compounds, including flavonoids and sulfur-containing molecules, have demonstrated efficacy in reducing inflammation-driven cochlear damage. Future research should focus on refining targeted anti-inflammatory therapies that selectively block pathological NF-κB activation while preserving its essential physiological functions.

### 4.2. Localized Delivery Strategies

#### 4.2.1. Intratympanic Delivery:

Intratympanic (IT) drug delivery has emerged as a promising approach for treating inner ear disorders while minimizing systemic exposure and potential interference with cisplatin’s antitumor effects [49,50]. This method allows for high drug concentrations in the inner ear while maintaining low systemic levels, thus reducing the risk of side effects and drug interactions [49]. Several studies have demonstrated the efficacy of IT administration for otoprotection against cisplatin-induced hearing loss. For instance, IT injection of N-acetylcysteine (NAC) resulted in very restricted systemic uptake while achieving substantially higher intracochlear drug levels compared to systemic administration [49]. Similarly, IT dexamethasone showed modest otoprotection against cisplatin ototoxicity without interfering with its chemotherapeutic effects [51]. However, there are some contradictions and challenges associated with IT delivery. While a 2% NAC concentration provided significant otoprotection, a 4% concentration resulted in reduced hearing capacity, highlighting the importance of optimal dosing [52]. Additionally, high-concentration NAC caused considerable inflammatory reactions, suggesting that not all formulations are suitable for IT administration [53]. In conclusion, IT drug delivery shows great potential for minimizing systemic exposure while maintaining cisplatin efficacy. It offers a targeted approach to otoprotection, allowing for high local drug concentrations with limited systemic effects. However, careful consideration must be given to drug formulations, concentrations, and delivery methods to optimize efficacy and safety. Further research is needed to refine IT delivery techniques and expand its applications in treating inner ear disorders [54].

#### 4.2.2. Nanoparticles and Hydrogels

Novel drug delivery systems for targeted release to the cochlea have shown significant promise in improving treatment efficacy for inner ear disorders. Nanoparticle-based approaches represent a major advancement, offering enhanced biocompatibility, stability, and cell/tissue uptake compared to traditional methods [55]. These nanocarriers can be designed for controlled and sustained release of therapeutic agents directly to specific cell types within the cochlea, maximizing therapeutic effects while minimizing systemic side effects [56]. Interestingly, microfluidics-based systems are emerging as a cutting-edge technology for cochlear drug delivery. These wearable or implantable microsystems provide improved control over pharmacokinetics and enable longer-term delivery, with potential applications in hair cell regeneration [57]. Additionally, cochlear implant-mediated drug delivery represents an innovative approach, combining electrical stimulation with localized drug administration [58]. Targeted drug delivery to the cochlea is rapidly evolving, with promising developments in nanoparticle-based systems, microfluidic technologies, and implant-mediated approaches. Novel biomaterials like chitosan-glycerophosphate hydrogels have also demonstrated sustained local drug release to the inner ear [59]. As research progresses, these advanced delivery systems have the potential to significantly improve treatment outcomes for a range of inner ear disorders, from ototoxicity to sensorineural hearing loss [60,61].

## 5. Emerging and Experimental Approaches

### 5.1. Genetic and Biomarker-Based Risk Stratification

Genetic predisposition, age, renal function, and comorbidities significantly influence the risk of ototoxicity in patients undergoing cisplatin treatment. Genetic association studies have identified several candidate genes that may contribute to cisplatin-induced ototoxicity. For instance, polymorphisms in genes such as *ACYP2*, *WFS1*, and *SLC16A5* have been linked to a higher risk of hearing loss following cisplatin therapy [62,63].

Beyond genetic markers, biomarkers play a crucial role in predicting patient susceptibility to ototoxicity and developing personalized otoprotection strategies. Genetic association studies have identified several candidate genes that may contribute to cisplatin-induced ototoxicity. The thiopurine methyltransferase (TPMT) gene is considered critical for susceptibility to cisplatin-induced hearing loss, as highlighted in a pharmacogenetic guideline [64]. Other genes of interest include ABCC3, OTOS, SLC22A2, NFE2L2, SLC16A5, LRP2, GSTP1, SOD2, WFS1, and ACYP2 [64]. Additionally, the APOE gene has been associated with biological age measures, which may indirectly influence ototoxicity susceptibility [65]. Interestingly, there are contradictions in the current research. While some studies have found promising genetic markers, others report low evidence due to small cohort sizes and contradictory replication studies [66]. The A1555G mitochondrial DNA mutation has shown a strong association with aminoglycoside ototoxicity, but evidence for other genetic variants remains limited [66]. Biomarkers for personalized otoprotection are an active area of research, with genetic markers showing potential for predicting patient susceptibility to ototoxicity. However, further studies with larger cohorts and consistent replication are needed to validate these findings. Future research should focus on developing a unified set of clinical, treatment, and genetic risk factors to create flexible models for forecasting post-treatment audiograms and improving patient care [67] (Table 2).

### 5.2. Novel Otoprotective Agents

Epigenetic modifications and non-apoptotic pathways are promising targets for cancer therapy and other diseases. Agents targeting DNA methylation and histone modifications have shown considerable potential in clinical studies for treating various cancers [71]. These epigenetic alterations significantly influence gene expression, cellular proliferation, differentiation, and apoptosis, making them attractive targets for therapeutic intervention. Epigenetic modifications, including histone modifications and DNA methylation, play crucial roles in regulating gene expression and can potentially modulate the expression of antioxidant defense genes in cochlear cells exposed to cisplatin. DNA methylation and histone modifications are known to influence gene expression in response to various environmental stresses, including oxidative stress [72]. Cisplatin, a chemotherapeutic agent, can induce oxidative stress in cells, potentially triggering epigenetic changes. Epigenetic modifications play a significant role in regulating genes involved in oxidative stress response, particularly SOD2 (superoxide dismutase) and GPX1 (glutathione peroxidase). In human multiple myeloma cells, the SOD2 gene encoding manganese superoxide dismutase (MnSOD) is epigenetically silenced through promoter hypermethylation. Treatment with the DNA methyltransferase inhibitor Zebularine reverses this methylation, increasing SOD2 gene expression and enzyme levels [73]. Similarly, in pulmonary artery endothelial cells, histone deacetylase (HDAC) inhibitors such as scriptaid and trichostatin A induce the expression of extracellular superoxide dismutase (EC-SOD) up to 10-fold. This induction is associated with increased histone H3 (Lys27) acetylation and H3 (Lys4) trimethylation at the gene promoter [74]. Interestingly, the regulation of oxidative stress-related genes can differ between antioxidant and pro-oxidant genes. While HDAC inhibitors increase EC-SOD expression, they simultaneously decrease the expression of the pro-oxidant gene NADPH oxidase 4 by more than 95% in pulmonary artery endothelial cells [74]. This differential regulation results in a significant reduction of cellular reactive oxygen species levels. Similarly, sound conditioning, a pretreatment using low-level non-damaging sound, has been found to protect against subsequent acoustic trauma by suppressing apoptotic pathways. This approach upregulates bcl-2, an inducible neuroprotective gene, and prevents the release of cytochrome c from mitochondria, thereby preserving hair cell function and hearing [75]. Consequently, it is an interesting alternative to traditional approaches for preventing noise-induced hearing loss. Targeting non-apoptotic pathways through epigenetic modifications or sound conditioning offers promising avenues for treating various diseases. Epigenetic drugs, such as DNA methyltransferase inhibitors and histone deacetylase inhibitors, have shown efficacy in clinical trials for cancer treatment [76,77]. Additionally, epigenetic alterations play a significant role in influencing apoptotic pathways in various cell types, including cochlear cells. While the provided context does not specifically address cochlear cells, we can draw insights from the general mechanisms of epigenetic regulation of apoptosis. Epigenetic modifications, such as DNA methylation, histone modifications, and non-coding RNAs, can significantly impact the expression of apoptosis-related genes, including BCL2 and TP53 [78]. These modifications can either promote or suppress cancer development by influencing various programmed cell death processes, particularly autophagy and apoptosis. In the context of BCL2, an anti-apoptotic gene, epigenetic alterations can lead to its overexpression, which has been associated with chemotherapy resistance in various human cancers (Kang & Reynolds, 2009) [79]. For instance, histone deacetylase inhibitors (HDACis) like vorinostat and romidepsin have been shown to selectively kill transformed cells by inducing a tumor-cell-selective pro-apoptotic gene expression signature, including the downregulation of the pro-survival gene BCL2A1 [80]. Regarding TP53, although not explicitly mentioned in the provided context, it is known to be a crucial pro-apoptotic gene. Epigenetic modifications can potentially influence its expression and activity, thereby affecting the balance between pro- and anti-apoptotic factors in cells. Table 3 shows the main agents and their effects.

Combination therapies have emerged as a promising approach for treating complex diseases by targeting multiple pathways simultaneously. This strategy aims to overcome the limitations of single-drug treatments and enhance therapeutic efficacy. Nanotechnology-based carriers have shown potential for sequential combination therapy, allowing for the controlled release of multiple drugs to target tumor cells [81]. These nanoformulations can sensitize tumor cells through cascaded drug delivery or can remodel the tumor microenvironment to enhance drug distribution. Similarly, in cardiac arrest treatment, a multi-drug cocktail targeting multiple pathways of ischemia-reperfusion injury has demonstrated improved neurologically survival rates in animal studies [82]. This approach addresses the complex metabolic disturbances following cardiac arrest, which single-drug therapies have failed to effectively target. Interestingly, the concept of polypharmacology has been successfully applied in HIV/AIDS treatment, with combined antiretroviral therapy (cART) providing life-saving benefits [83]. However, the development of new drugs remains crucial due to emerging resistant strains and poor adherence to cART. In the context of Alzheimer’s disease, a multitargeted anti-aging approach using drug cocktails has been proposed to address the complex age-related mechanisms involved in the disease progression [84]. The development of multi-drug cocktails offers a promising strategy for comprehensive protection against complex diseases. However, optimizing these combinations remains challenging. Advanced techniques such as microfluidic systems for precise drug dispensing [85] and information-theoretic active learning approaches [86] may help streamline the process of identifying effective drug combinations. Further research and clinical validation are needed to fully realize the potential of these multi-targeted therapies.

### 5.3. Statin–Cisplatin Interactions and Their Implications for Ototoxicity-Reducing Therapies in Cancer Treatment

Cisplatin exerts its ototoxic effects primarily by inducing reactive oxygen species (ROS) generation, leading to oxidative damage in cochlear hair cells and spiral ganglion neurons. Excess ROS triggers mitochondrial dysfunction, activating intrinsic apoptotic pathways that result in progressive hearing loss [27,87]. Statins, particularly pravastatin and atorvastatin, have demonstrated antioxidant properties, enhancing the activity of key antioxidant enzymes such as superoxide dismutase (SOD) and glutathione peroxidase (GPX), which help scavenge ROS and reduce oxidative damage [88].

In addition to oxidative stress, inflammation plays a critical role in CIO. Cisplatin activates the NF-κB signaling pathway, leading to the upregulation of pro-inflammatory cytokines such as TNF-α, IL-6, and COX-2, which exacerbate cochlear damage [89,90]. Statins have been shown to suppress NF-κB activity, reducing inflammation-mediated hair cell degeneration. Pravastatin, in particular, has demonstrated downregulation of inflammatory cytokines in models of cardiovascular and renal injury, suggesting a potential protective role in the cochlea [90].

Mitochondrial dysfunction and apoptosis further contribute to CIO, with p53 upregulation playing a key role in promoting cisplatin-induced programmed cell death. Studies have shown that pravastatin can suppress p53 expression, thereby inhibiting mitochondrial-dependent apoptosis and preserving cell viability in cisplatin-treated tissues [91]. Given that mitochondrial dysfunction is a major contributor to hearing loss, pravastatin’s ability to modulate apoptotic pathways may offer substantial otoprotective benefits.

Finally, cochlear ischemia due to endothelial dysfunction is another key contributor to CIO. Cisplatin impairs endothelial nitric oxide synthase (eNOS) activity, reducing cochlear blood flow and exacerbating ischemic damage. Pravastatin and atorvastatin have been shown to enhance nitric oxide bioavailability, improving microvascular perfusion and oxygen delivery, which may help maintain cochlear function during cisplatin therapy [92].

Several studies have investigated the efficacy of statins in mitigating CIO. Lovastatin has been found to protect against cisplatin-induced hearing loss in adult mice, with greater protection observed in females compared to males, suggesting that sex-based differences may influence statin efficacy (Fernandez et al., 2020) [8]. Atorvastatin users experienced a significantly lower incidence of hearing loss (9.7%) compared to non-statin users (29.4%) during cisplatin treatment, further supporting statins’ potential role in otoprotection [93].

Despite these promising findings, clinical studies on statins as otoprotective agents remain inconclusive. Some trials have reported no significant differences in hearing loss rates between statin users and non-users, highlighting variability in efficacy (McCullough et al., 2022) [90]. The inconsistency in findings underscores the need for extensive clinical trials to validate these results and optimize dosing regimens to ensure maximum benefit without interfering with cisplatin’s chemotherapeutic activity [92].

One major concern regarding otoprotective agents is the potential for interference with cisplatin’s antitumor effects. Unlike many antioxidant therapies, statins have demonstrated anticancer properties, including inhibition of tumor growth, induction of apoptosis, and suppression of metastasis [94].

## 6. Clinical Implications

Biomarker-based risk assessment holds promise for tailoring protective interventions against cisplatin-induced ototoxicity (CIO). Genetic variants contributing to CIO have been identified through genome-wide association studies, highlighting the polygenic nature of this adverse drug reaction [95]. This genetic information can be leveraged to develop polygenic scores (PGSs) for predicting CIO risk, potentially enabling more targeted prevention efforts. A study demonstrated the development of a biologically informed PGS for CIO (PGSCIO) using large-scale hearing loss genome-wide association study data combined with single-cell RNA sequencing data from cisplatin-treated murine inner ears. This PGSCIO showed superior performance in predicting CIO risk compared to a general hearing loss PGS, suggesting its potential for identifying high-risk individuals [95]. Such biomarker-based risk assessments could guide the implementation of personalized protective strategies. Interestingly, while personalized medicine approaches show promise, current interventions for preventing CIO in adult cancer patients have shown only mild benefits. Sodium thiosulfate appears to be the most promising preventive strategy, but there is still a need for more rigorous, high-quality research to evaluate potential symptoms and innovative treatment modalities [13]. The integration of genetic risk assessment with other clinical factors could lead to more effective, tailored interventions. For instance, considering that one in five adult cancer patients treated with cisplatin develops symptomatic high-frequency hearing loss [96], combining genetic risk scores with clinical risk factors could help identify those most likely to benefit from protective interventions. In conclusion, biomarker-based risk assessment, particularly using polygenic scores, offers a promising approach for tailoring protective interventions against cisplatin-induced ototoxicity. By identifying high-risk individuals, clinicians could implement targeted prevention strategies, potentially including pharmacological interventions like sodium thiosulfate or other emerging therapies. However, further research is needed to translate these genetic insights into clinically actionable interventions and to evaluate their effectiveness in preventing or mitigating cisplatin-induced ototoxicity. A team-based approach involving oncologists, audiologists, and nurses is crucial for effectively managing ototoxicity in cancer patients: Oncologists play a central role in ototoxicity management as the primary source of information for patients about the potential ototoxic effects of chemotherapy [97]. They are responsible for considering treatment modifications if alternative options are available to reduce ototoxicity risks [98]. However, studies have found that oncologists’ awareness and practices regarding ototoxicity monitoring vary widely, with some routinely referring patients for audiological evaluation while others rely on patient self-referral [98,99]. Audiologists are essential team members for implementing ototoxicity monitoring programs and providing specialized hearing assessments [97,100]. Their involvement allows for the early identification of hearing loss and timely intervention. However, the role of audiologists is not always fully realized within oncology teams, and there is often a lack of established protocols for ototoxicity monitoring in hospitals [97,101]. Nurses, especially oncology nurses, play a critical role in patient care and education. However, studies have found varying levels of awareness about ototoxicity among nursing staff, highlighting the need for improved education and training [97,99]. Interestingly, some studies advocate for an expanded multidisciplinary approach, including pharmacists in the ototoxicity monitoring team. [100,102] This interdisciplinary collaboration can lead to more effective service delivery and patient management. In conclusion, while a team-based approach is recognized as crucial for managing ototoxicity [103], implementation remains challenging. There is a need for improved awareness, established protocols, and better integration of audiological services within cancer care teams to enhance ototoxicity monitoring and management [99,104]. Future strategies should focus on fostering collaboration among team members and standardizing ototoxicity monitoring practices across healthcare facilities.

Healthcare providers play a crucial role in managing ototoxicity and implementing protective strategies. Training in this area is essential for several reasons: Firstly, effective ototoxicity management requires a multidisciplinary approach involving audiologists, clinical pharmacists, and physicians [103,105]. Healthcare providers need to be aware of the potential effects of ototoxic medications and be able to identify patients at increased risk of developing ototoxicity [105]. This knowledge is critical for implementing appropriate administration and monitoring protocols to prevent auditory impairment. Interestingly, despite widespread recommendations for early and effective ototoxicity monitoring and management, there is limited evidence of implementation in healthcare services across the UK [106]. About 72% of surveyed centers reported the absence of ototoxicity management protocols, highlighting a significant gap in provider education and practice guidelines [106]. To address these educational needs, healthcare providers should be trained in several key areas. These include understanding ototoxic medications and their effects, implementing pharmacological and audiological monitoring strategies, and utilizing otoprotective substances where appropriate [103,105]. Additionally, providers should be educated on the importance of proactive ototoxicity management strategies, which aim to minimize exposure, avoid symptom onset, provide ongoing monitoring, and manage auditory and vestibular changes as patients’ clinical needs evolve [107]. By improving education and awareness in these areas, healthcare providers can better contribute to preserving patients’ hearing and vestibular function while effectively treating life-threatening conditions [103].

## 7. Conclusions

Even if cisplatin shows good anti-cancer response, one important disadvantage is ototoxicity. Ototoxicity can appear due to oxidative stress induced by cisplatin. It is necessary to develop different technologies and techniques that can have an anti-cancer effect, but also show a protective effect against ROS. Antioxidants and anti-inflammatory substances showed promising results for anti-cancer effects and ROS protection, but further research is necessary to establish the combination and dosage for the best results. Apoptosis inhibitors such as XIAP, L-NAC, and Apaf-1 inhibitors have shown promise in reducing cochlear cell death and protecting against hearing loss caused by toxic agents like gentamicin and cisplatin. These findings show the therapeutic potential of targeting apoptotic pathways to prevent hearing loss, especially in ototoxicity treatment. Interesting findings show that epigenetic modifications and non-apoptotic pathways present new avenues for treating various diseases, including cancer and hearing loss. Approaches like sound conditioning and epigenetic drugs have shown potential in preventing hearing loss and modulating gene expression. These novel therapies offer new possibilities for targeting complex disease mechanisms. Also, pravastatin’s potential goes beyond its decreasing cholesterol effects, with evidence suggesting benefits in reducing cardiovascular risks and protecting against nephrotoxicity. Its antioxidant properties and impact on cellular stress responses, particularly through modulation of the p53 pathway, make it a promising candidate for diverse therapeutic applications. Further studies are needed to fully explore its potential across various clinical scenarios.

## Figures and Tables

**Table 1:** Summary of mechanisms of cisplatin-induced ototoxicity, pathways, key studies, and therapeutic implications.

Mechanism	Pathway/Details	Key Studies	Therapeutic Implications
Oxidative Stress	ROS generation causing lipid, protein, and DNA damage; activation of apoptotic pathways.	[16,17,18]	Antioxidants like NAC and vitamin E could neutralize ROS.
Apoptosis	Activation of JNK and p38 MAPK pathways, leading to hair cell apoptosis.	[20]	Caspase inhibitors and agents targeting JNK/MAPK pathways.
Necroptosis	Involves RIPK1 and RIPK3; a programmed necrosis contradicting earlier views of passive necrosis.	[21]	Necroptosis inhibitors targeting RIPK1 and RIPK3 pathways.
Ferroptosis	Iron-dependent lipid peroxidation; recently linked to cochlear cell death in cisplatin ototoxicity.	[22]	Potential use of ferroptosis inhibitors in preclinical research.
Autophagy	Dual role: cytoprotective effects early, but later induction of cell death via mTOR suppression.	[23]	Agents modulating autophagy pathways may reduce cell death.
Nitrative Stress	S-nitrosylation of cochlear proteins; peroxynitrite formation linked to cellular damage.	[28]	Inhibitors of nitrative stress, such as Trolox, show potential.
Parthanatos	PARP-1-dependent cell death; linked to oxidative stress and cochlear cell damage.	[22,23]	Targeting PARP-1 pathways could mitigate ototoxic effects.

**Table 2:** Genetic polymorphisms associated with cisplatin-induced ototoxicity.

Gene	Polymorphism	Associated Risk	Study Findings	References
ACYP2	rs1872328	Increased risk of hearing loss	Polymorphism linked to higher susceptibility to cisplatin-induced hearing loss in pediatric patients	[62]
WFS1	rs62283056	Increased risk of hearing loss	Dose-dependent effect; individuals with the minor allele exhibit greater hearing loss at higher cumulative cisplatin doses; replicated in a cohort of 18,620 patients	[68]
SLC16A5	rs4788863	Protection against hearing loss	Identified as protective against cisplatin-induced ototoxic effects in two independent cohorts. Functional validation revealed that SLC16A5 silencing altered cellular responses to cisplatin, suggesting its role in ototoxic mechanisms. Previous studies highlight potential otoprotective strategies using SLC16A5 inhibitors like cimetidine.	[63]
TPMT	rs12201199	Increased risk of hearing loss	Genetic variant associated with cisplatin-induced hearing loss in children. Found in two cohorts (*p* = 0.00022); functional validation suggested significant susceptibility.	[69]
COMT	rs9332377	Increased risk of hearing loss	Genetic variant linked to higher susceptibility to cisplatin ototoxicity in pediatric patients; association replicated (*p* = 0.00018).	[70]

**Table 3:** Overview of epigenetic modifications, sound conditioning, and combination therapies with their therapeutic potential and key studies.

Approach	Description	Key Studies	Therapeutic Potential
Epigenetic Modifications	Targets DNA methylation and histone modifications to influence gene expression and apoptosis.	[71,76,77]	Epigenetic drugs show efficacy in cancer treatment by modulating cellular processes.
Sound Conditioning	Uses low-level non-damaging sound to upregulate neuroprotective genes (e.g., bcl-2) and prevent cytochrome c release.	[75]	Promising alternative for preventing noise-induced and other types of hearing loss.
Combination Therapies	Targets multiple pathways simultaneously to enhance therapeutic efficacy and overcome single-drug limitations.	[81,82]	Addresses limitations of single-drug therapies; applicable in cancer, cardiac arrest, and HIV/AIDS.
Nanotechnology-based Carriers	Employs nanocarriers for controlled drug release, tumor sensitization, and improved drug distribution.	[81,82]	Improves bioavailability and targeted drug delivery with minimized side effects.
Multi-drug Cocktails	Combines multiple drugs to address complex diseases; used in HIV/AIDS, cardiac arrest, and Alzheimer’s.	[83,84]	Provides life-saving benefits and addresses multiple disease mechanisms; requires optimization.
